# The Comparison Between the Different Types of Storage Mediums on the Viability of Periodontal Cells Prior to the Replantation of Avulsed Teeth: A Systematic Review & Meta-Analysis

**DOI:** 10.3390/jcm14061986

**Published:** 2025-03-14

**Authors:** Charlotte Anne Blackledge, Marcela Ferrer Molina, Tawfiq Hijazi Alsadi, Susana Muwaquet Rodriguez

**Affiliations:** 1 Department of Dentistry, Universidad Católica de Valencia, 46001 Valencia, Spain; charblac@mail.ucv.es; 2 Department of Orthodontics, Faculty of Medicine and Health Science, Catholic University of Valencia, 46001 Valencia, Spain; marcela.ferrer@ucv.es (M.F.M.); tawfiq.hijazi@ucv.es (T.H.A.); 3 Department of Restorative Dentistry and Endodontics, Faculty of Medicine and Health Science, Catholic University of Valencia (UCV), C/Quevedo, 2, 46001 Valencia, Spain

**Keywords:** avulsed permanent teeth, Hank’s balanced salt solution, in vitro, periodontal cell viability, storage media

## Abstract

**Background/Objectives**: Dental avulsion involves the complete displacement of the tooth from its socket and falls into the most severe categories of the various types of traumatic dental injuries. Successful replantation of the tooth depends on various factors such as the time between the event and replantation, as well as the extra-alveolar storage medium and duration. The adoption of the correct handling measures and use of an appropriate storage medium are key factors that affect the long-term prognosis of the avulsed tooth following replantation. This systematic review and meta-analysis aim to determine if Hank’s Balanced Salt Solution (HBSS) is the most effective storage medium to preserve periodontal (PDL) cell viability following avulsion. **Methods**: A comprehensive review of the literature available was conducted on the 18 November 2024 across three databases, namely EBSCO (including PubMed-Medline), Scopus, and Web of Science. The review was written according to and following the Preferred Reporting Items for Systematic Reviews and Meta-Analyses (PRISMA) statement created in 2009 and updated in 2020. The following PICO question was constructed for the systematic review: “In patients with avulsed permanent teeth, is Hank’s balanced salt solution more effective in preserving periodontal cell viability to increase the likelihood of a more successful replantation than any other storage media technique available?” A meta-analysis was also conducted with the selected studies, and the software used for this was R 4.3.1 (R Core Team (2018)). **Results**: A total number of 443 articles were found in the initial search. Of these 443, 9 articles were included in the final systematic review and meta-analysis. 6 out of the 9 articles conclude HBSS to be the most superior storage medium for the preservation of the PDL cells, whilst the other three concluded PDL cell preservation was higher in Morinda citrifolia juice, propolis, and coconut water, suggesting a suitable alternative to HBSS. Nonetheless, the meta-analysis indicated that PDL cell viability was significantly higher using HBSS compared to all other storage media. **Conclusions**: The systematic review and meta-analysis have provided adequate data in favor of the alternative hypothesis, indicating that Hank’s balanced salt solution is the most effective storage medium in the preservation of periodontal cell viability following the avulsion of permanent teeth.

## 1. Introduction

Dental avulsion, also termed total luxation or exarticulation, can be defined as the “total displacement of the tooth out of its socket” [1]. The avulsion of the permanent teeth is seen in 0.5–16% of all dental injuries, often following sporting injuries, with the maxillary central incisors being the most frequently involved tooth, followed by the maxillary lateral incisors. When a tooth is subjected to avulsion, its whole structure is significantly disrupted. As the tooth becomes separated from its socket during avulsion, the periodontal ligament fibers become torn and severed, and this leaves viable periodontal ligament (PDL) cells on the root surface. The maintenance of the viability of these cells is essential for long-term retention of the eventually replanted teeth and depends on the appropriate selection of an appropriate storage medium and extra-alveolar period and handling [2]. There are three key parameters that can influence the clinical efficacy of the storage medium: osmolarity or pH, extra-alveolar duration, and temperature [3]. The longer the tooth remains outside of the mouth results in fewer chances of PDL healing and higher chances of root resorption. In addition, if five minutes pass without placing the tooth in some kind of solution or medium, the effects can significantly impact the regeneration of a normal PDL, decreasing the clonogenic and mitogenic capacity of the PDL cells [3]. Therefore, the selection of a medium with optimal osmolarity in the range of 230 to 400 mOsm/L is essential for the survival and maintenance of normal cell growth [4]. In addition to these parameters, the chosen storage medium should encompass various properties to reduce the potential harmful effects upon the PDL cells. Such ideal properties include the ability to maintain PDL cell viability, contain a physiological pH and osmolarity, have clonogenic and mitogenic capacity, not be capable of inducing an antigen–antibody reaction, have antimicrobial properties, be effective under different conditions, and lastly, to be readily and easily available/accessible [5].

Hank’s Balanced Salt Solution (HBSS) is considered the “gold standard” amongst the various media available as it has the ability to maintain the vitality of the proliferative capacity of the PDL cells. HBSS creates an optimum environment for cell survival and cell growth as its osmolarity falls within the optimal range of 320 mOsmol and has a pH of 7.2. In addition to this, it has been proven to be the most successful medium in the maintenance of the mitogenicity and clonogenic potential of PDL cells [6]. HBSS has even been accepted by the American Academy of Endodontics as a suitable medium for avulsed teeth due to its ability to maintain the vitality and proliferative capacity of PDL cells for a period of up to 48 h [3]. However, HBSS is not always available, and it is not a common solution to find at sites where TDIs occur. This is why it is important to establish and investigate other storage medium alternatives for when emergency situations occur. Alternative storage media such as aloe vera have shown promising results, with research indicating that is has the ability to maintain the integrity of the PDL fibers given its high water content, antioxidant and anti-inflammatory properties, making it a potentially attractive and cost-efficient option in emergencies [7]. Similarly, platelet-rich fibrin, which releases growth factors to promote healing, has gained recent attention for its potential in supporting PDL regeneration and improving tissue repair after replantation [8], but given its limited access and availability, its use in emergency situations is limited. Alternatively, solutions like coconut water, propolis, and coconut milk have also been shown to have potential; however, despite their beneficial properties, they also come with limitations. The acidic pH found in coconut water damages cell metabolism [9], while the high fat content in coconut milk has been seen to reduce cell viability [10]. Other readily available solutions such as saline and Ringer’s lactate are effective as short-term storage medium only, due to their limited amount of essential nutrients, which are essential for cell survival [11,12,13]. In addition, milk can be used as another viable short-term option, but it lacks the ability to support the metabolic needs of the PDL cells [14]. Egg albumin, a solution derived from egg whites, has been suggested as a preferable choice over that of milk given its high water and protein content along with its optimal pH of 8.6 to 9.3 and osmolarity of 258 mOsmol [11], providing results of PDL cell viability equivalent to those found when using HBSS [15]. Other promising media, such as Ocimum sanctum extract, contain antioxidant properties that can promote collagen formation and osteogenic differentiation, resulting in optimal wound healing [16]. Additionally, Morinda citrifolia juice has been shown to have promising effects on the regeneration of bone and periodontal tissues, suggesting its potential as an osteoinductive agent [16]. However, what is more surprising is that studies have discovered its potential to replace HBSS as a medium for the storage of avulsed teeth, as it has shown to preserve cell viability with results exceeding those found in HBSS [17].

As such, this systematic review and meta-analysis was conducted to explore the effectiveness of other types of storage media in the preservation of PDL cell viability that could be used as an alternative when HBSS is not available. Through this comparison, the results can not only help dental professionals provide evidence-based recommendations for emergency situations where HBSS is not obtainable, but they can also identify the most effective alternatives to ensure the best possible prognosis for the patient.

## 2. Hypothesis

This study hypothesizes that HBSS is the most effective storage medium in the preservation of PDL cell viability in avulsed permanent teeth prior to replantation. The null hypothesis states that HBSS does not significantly outperform other storage media, while the alternative hypothesis suggests that HBSS maintains a higher number of viable PDL cells compared to alternative solutions.

## 3. Objectives

The objective of this systematic review and meta-analysis is to analyze the effectiveness of HBSS and different types of storage mediums in maintaining periodontal ligament cell viability for avulsed permanent teeth prior to their replantation. The meta-analysis aims to provide a comprehensive conclusion regarding the most effective storage medium for preserving PDL cell viability via information gathered from different in vitro studies.

More specifically, this review seeks to identify the other types of storage media that may serve as viable alternatives to HBSS, assessing their relative effectiveness in preserving PDL cells from avulsed permanent teeth. These findings will help to inform dental professionals regarding evidence-based recommendations in the management of dental avulsion, particularly in emergency situations where HBSS may not be readily available.

### 3.1. Materials and Methods

This systematic review was formulated by following the statement of the PRISMA Guide (referring to “Preferred Reporting Items for Systematic Reviews and Meta-Analyses”). A comprehensive electronic search was carried out on the 18 November 2024 across three databases seen in the results section of this review. The search strategy involved using Medical Subject Headings (MeSH) terms and Boolean operators in combination with keywords according to the articulated PICO question. The following structure was used to formulate the PICO question for the review:

P (Population): Patients with avulsed permanent teeth.
I (Intervention): Storage of the avulsed tooth in HBSS prior to replantation.
C (Comparison): Storage of the avulsed tooth in other media, in particular saline, milk, Ocimum sanctum extract, Morinda citrifolia juice, Ringer’s lactate, egg albumin, coconut milk, platelet-rich fibrin, aloe vera, coconut water, propolis, pomegranate juice, and placentrex.
O (Outcome): The most effective storage medium to maintain periodontal ligament cell viability prior to replantation.

Thus, the PICO question was as follows: “In patients with avulsed permanent teeth, is Hank’s balanced salt solution more effective in preserving periodontal cell viability to increase the likelihood of a more successful replantation than any other storage media technique available?”.

### 3.2. Eligibility Criteria

The eligibility criteria for the studies included adherence to the following conditions stated below in Table 1 and Table 2.

### 3.3. Search Strategy and Study Selection

As mentioned previously, a comprehensive electronic search was carried out on the 18 November 2024, using three main databases: EBSCO, Web of Science, and Scopus. Using the relevant MeSH terms and Boolean operators in combination with the keywords selected alongside the constructed PICO question and the application of the aforementioned exclusion criteria, the exact search conducted in each database can be seen in Table 3 below.

The keywords inserted in each database were the same and included “avulsed permanent teeth”, “periodontal cell viability”, “hanks balanced salt solution”, “storage media”, and “in vitro”. Additionally, the Boolean operators “AND” or “OR” were used alongside the formulated PICO question. The filters applied to the searches included articles published within the last 10 years (2014–2024) and publications in the English language.

The search across all three databases was as follows: ((((avulsed permanent teeth) AND (periodontal cell viability)) AND (hanks balanced salt solution)) OR (storage media)) AND (in vitro). These searches were made individually across each of the databases with the objective of retrieving the most relevant and suitable articles regarding storage media for avulsed permanent teeth.

### 3.4. Data Extraction

Table 4, illustrated below, was fabricated to highlight the information and data that were relevant and, as such, extracted from the studies. It included information regarding the title and authors of the articles, year of publication, the type of study conducted, the number of teeth that were tested, the types of storage media, any complications that arose during the investigations, and lastly, the most recommended storage solution for the preservation of the PDL cells concluded in each article.

### 3.5. Data Synthesis

For the meta-analysis, the data that was extracted from the chosen studies included the standard deviation, mean, and number of teeth involved in each of the studies. The results provided are for both storage in HBSS and the other control groups to facilitate the comparison between their effectiveness on periodontal cell viability.

A meta-analysis was carried out to evaluate the data that was extracted in order to compare the different outcomes across the selected studies, in particular to assess the effectiveness of HBSS on preserving periodontal cell viability compared to other storage media. The researchers conducted an extensive review of the literature, with the final number of studies totaling 10. However, one of the studies was removed and excluded due to insufficient statistical data; therefore, only nine of the studies provided sufficient information and data to be included in the meta-analysis. In addition, from the review of the literature, the researchers decided that only two comparisons for the meta-analysis would be possible: HBSS vs. other (nine studies), and HBSS vs. aloe vera (four studies).

The primary objective of the meta-analysis was the assessment of PDL cell viability following avulsion and subsequent storage. Significant heterogeneity across the final studies was found because the variables and units of measurement were not uniform across all the studies. For example, particular studies compared HBSS with other storage media, and others compared HBSS with aloe vera. Additionally, although standard deviations (SD) and mean values were reported in the majority of the studies, it was found that when several storage media were assessed, these values were averaged for the sake of simplicity. Thus, results were presented as standard mean differences (SMD) to allow for the variability in the various measurement scales used in the studies. Therefore, the calculated SMD was used as a measure of differences in PDL cell viability amongst HBSS and other storage media. To analyze the data, a random-effect model was used with 95% confidence intervals (CI).

However, in order to account for the variabilities in the study designs, a heterogeneity analysis was performed. This included the calculation of an I^2^ index of heterogeneity and Cochran’s Q test, both revealing the presence of high heterogeneity, implying limitations of considerable variability among the studies investigated. Moreover, to assess the potential for publication bias, a funnel graph was prepared, and Egger’s test was applied to contrast this hypothesis. The significance level used in the analyses was 5% (α = 0.05). The software used to conduct this meta-analysis was the metafor package from R 4.3.1 (R Core Team (2018)). R: A language and environment for statistical computing. R Foundation for Statistical Computing, Vienna, Austria. URL: http://www.R-project.org/).

### 3.6. Quality and Risk of Bias Assessment

Overall, the reports that were included in this systematic review were 9 in vitro studies due to the nature of the interventions on the condition studied (PDL cell viability). As such, in order to assess the evidence, the modified CONSORT checklist was used to report the quality of laboratory studies, in particular in vitro studies, and to assess the risk of bias. In addition, the PRISMA statement was used to assess the quality of the systematic reviews.

## 4. Results

From the initial search, a total of 443 articles were obtained from the mentioned databases: Web of Science (*n* = 334), EBSCO (*n* = 79), and Scopus (*n* = 30). After having carried out the initial search, the next step involved was to remove duplicated records prior to screening, which was carried out manually, and a total of 39 duplicates were found and subsequently removed. Next, a preliminary screening was carried out, which involved reading the article titles, and those that did not meet the inclusion criteria or were not relevant to the topic were removed. This equated to the removal of 326 articles in total. Following this, 71 records were sought for retrieval, which involved reading the abstract of the article that provided an overview of the introduction, materials and methods, results, and conclusion of the selected article. After having read these 44 articles, they were removed as they were considered irrelevant, or they did not answer the PICO question. Afterwards, a more in-depth assessment of the remaining 27 articles was carried out, which involved a thorough and extensive reading of the whole article text. Those articles that were not eligible for the analysis were removed and excluded. The reasons for the exclusion of these articles were due to three of them being systematic reviews, which was not realized until the in-depth reading of the work; another three studies were conducted on animals that made up part of the exclusion criteria; three other studies conducted investigations on other variables that did not include PDL cell viability following storage, namely these were the effect of temperature, clonogenic capacity, and cell apoptosis. In addition, one study did not provide any data on cell viability following storage, and another study had to be excluded because it was an ex vivo experiment. The last seven studies excluded were due to the fact that HBSS was not used as a storage medium in the studies.

**Figure 1:**
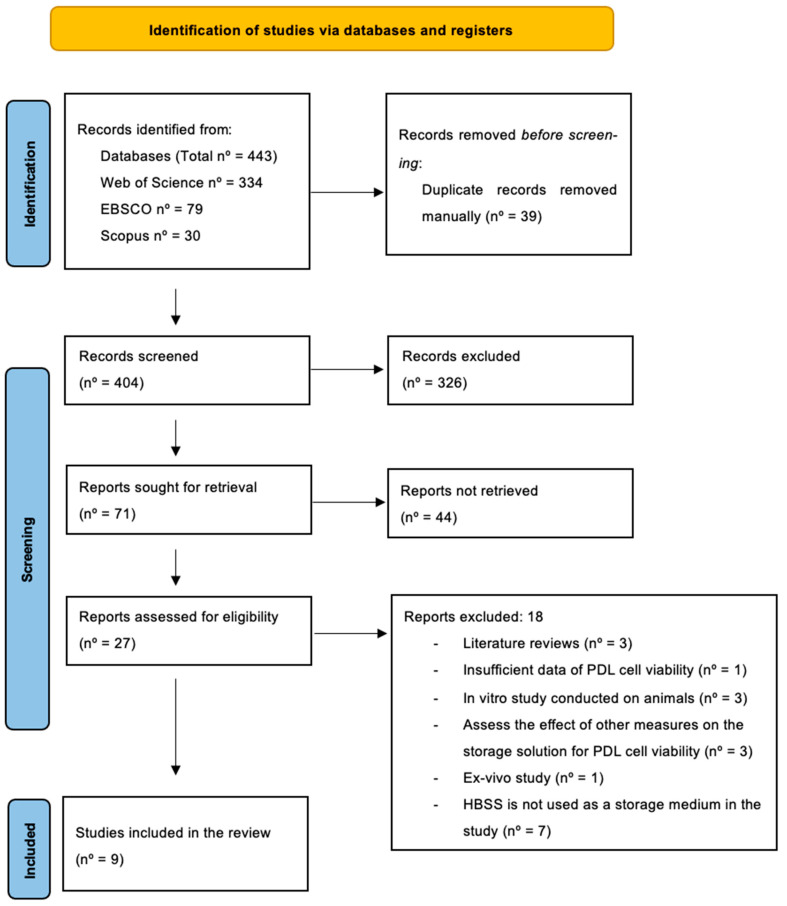
PRISMA flowchart demonstrating the scheme that was followed in the selection of articles.

The selection process of the studies therefore results in a total of nine articles that were eligible following screening. This process is outlined below in Figure 1.

The primary aim of the research is to assess the influence of the storage medium on the preservation of the tooth following avulsion. The review and combination of results of several studies are conducted using meta-analysis. The researcher looks to integrate the information derived from the different reports to reach a general conclusion. Out of the final selection of ten articles reporting on the primary outcome of the number of viable PDL cells, only nine provided mean ± SD. As such, only one (Esber) did not and was therefore excluded from the analysis. All articles are two- or multiple-arm comparisons of different storage media. Hank’s balanced salt solution (HBSS) was the medium that was compared with other different solutions. Most of them have a low presence, but only ‘aloe vera’ was used in four studies. In order to appropriately assess the influence of storage media on the preservation of the tooth following avulsion, the meta-analysis is divided into two parts. First is the comparison between HBSS as a storage medium vs. others (model 1), and then secondly, the comparison between HBSS vs. aloe vera only (model 2). The total number of patients (teeth) was *n* = 416:123 teeth were stored using HBSS and 293 using ‘other’ solutions.

In model 1, the information for the final input of the meta-analysis data can be seen below in Table 5. An initial overview of the table results reveals a higher PDL count (mTX) in the HBSS group compared to the control group. Additionally, some studies report extremely high results for PDL counts, such as Shetty and Abraham (2019) [20], which can indicate differences in methodology/reporting resulting in variability of results.

**Figure 2:**
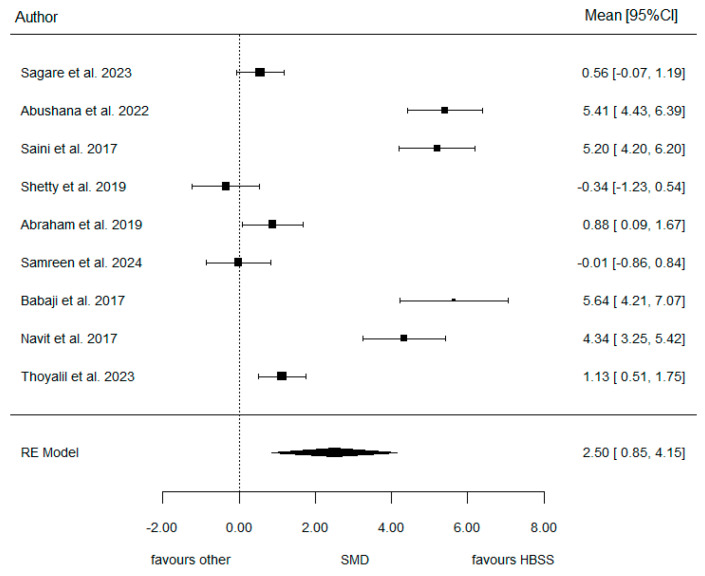
Forest plot for model 1 [9,15,16,19,20,21,22,23,24].

Additionally, the forest plot (seen in Figure 2 below) graphically displays the estimated results of the meta-analysis. Studies from Abushana et al. (2022) [18], Saini et al. (2017) [10], and Babaji et al. (2017) [22] show a strong preference for HBSS as their SMD (standardized mean difference) values are significantly positive with narrow confidence intervals. On the other hand, other studies, such as those by Shetty et al. (2019) [19] and Samreen et al. (2024) [21], show near-zero or slightly negative values, suggesting no strong preference or favoring of other media. However, the RE Model (random effects model) provides an overall pooled estimate of 2.50 (0.85, 4.15) indicating that HBSS generally outperforms other media. As such, the meta-analysis concluded the SMD to be 2.50, representing the standardized difference in PDL counts between HBSS and other groups (seen in Table 6 below). One must bear in mind that it is not interpreted in terms of units but rather the effect size between both groups. A positive value is interpreted as a higher PDL count found in the HBSS group. The 95% confidence interval for this overall effect measure (0.85–4.15) does not cross 0, indicating a strong statistically significant trend (*p*-value = 0.003), confirming the hypothesis that HBSS significantly outperforms other solutions in the preservation of PDL cells. Table 6 also demonstrates a high level of heterogeneity (I^2^ = 97%). The possible causes of the high heterogeneity can be due to the variability among the studies. For example, some studies had smaller sample sizes, leading to larger variability, such as studies by Shetty et al. (2019) [19], which had a sample size of 20, and Samreen et al. (2024) [21], with 24, compared to all the other studies, which had sample sizes ranging between 30 and 80. In addition, high heterogeneity can be interpreted due to differences in the tooth types used and the reasons for extractions. This can be seen in the study by Shetty et al. (2019) [19], who performed the extraction of molars for orthodontic or periodontal reasons, compared to the other studies that involved the extraction of premolars for only orthodontic reasons.

**Figure 3:**
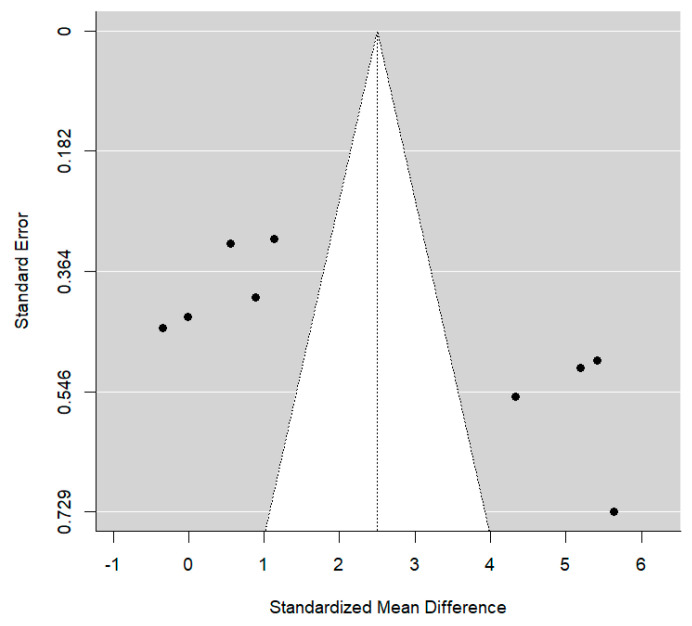
Funnel plot for bias of publications in model 1.

The presence of publication bias is shown in the funnel plot below (Figure 3). The four studies by Abushana, Saini, Babaji, and Navit are located in the lower right part of the plot due to the standard error associated with their SMD being larger. They were less precise studies compared to the rest.

**Figure 4:**
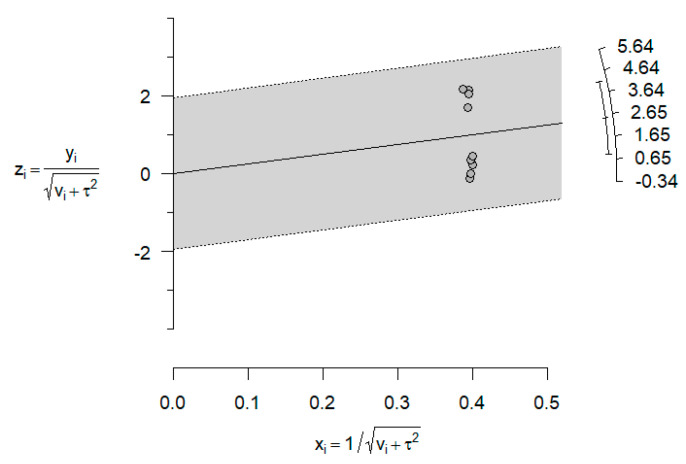
Galbraith’s plot for model 1.

Looking at the previous forest plot above, in Figure 2, we detect some authors (Abushana, Saini, Babaji, Navit) involving large effect sizes (mean differences relativized to SD) favoring strongly HBSS against the rest with some moderate or null effects. Observe that the difference is visualized in the Galbraith’s plot below (Figure 4). There are no individual articles, especially near the confidence bounds, but an overall separation of two blocks of articles.

The final input of the data included in model 2, which compared HBSS only with aloe vera, can be seen below in Table 7. From the initial overview of the table, we can observe that the SD from the different articles was not reasonably comparable.

**Figure 5:**
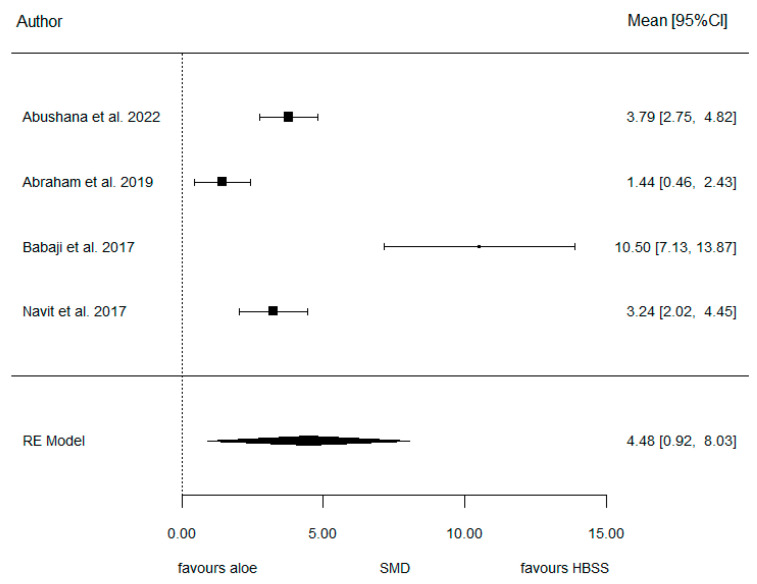
Forest plot for model 2 [15,20,22,23].

Moreover, the forest plot below for model 2 (Figure 5) graphically displays the estimated results of the meta-analysis. The meta-analysis concluded the SMD to be 4.48 in this case (95% CI: 0.92, 8.03), which again does not cross 0, therefore indicating a strong statistically significant trend (*p*-value = 0.014). This significance also confirms that HBSS provides significantly better preservation than aloe vera.

**Figure 6:**
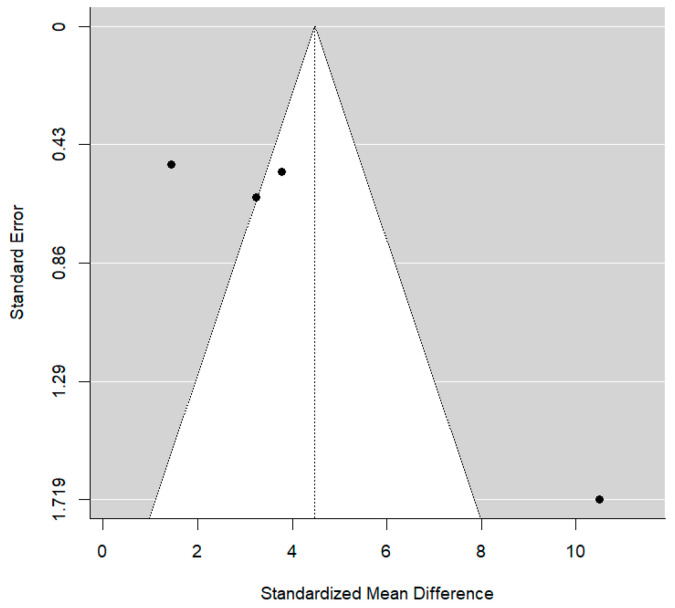
Funnel plot for model 2.

**Figure 7:**
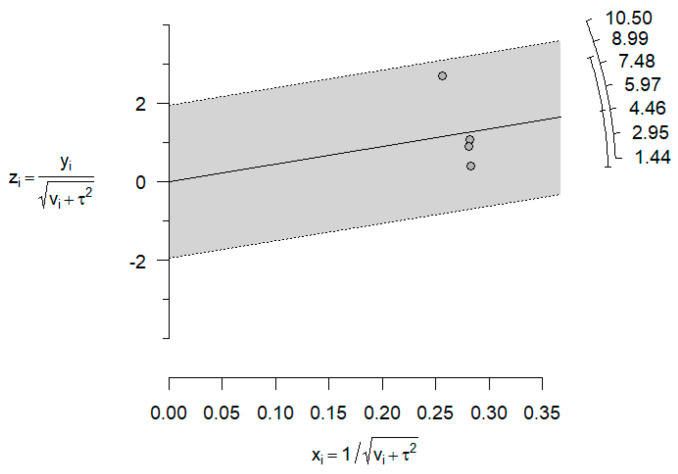
Galbraith’s plot for model 2.

Again, the heterogeneity, observed in Table 8, was estimated at a high level of 96.7%, with Babaji (2017) [22] showing a benefit of HBSS that is very large compared to other authors. Additionally, the absence of publication bias was rejected (*p* > 0.001) as seen in the funnel plot (Figure 6) below for model 2. Additionally, the Galbraith’s plot seen in Figure 7 suggests a slight trend in effect sizes, but the confidence band includes zero, indicating that whilst some heterogeneity may exist, there is no strong evidence of significant small-study bias in the meta-analysis.

## 5. Discussion

The general objective of the systematic review was to analyze the effectiveness of HBSS and different types of storage mediums in maintaining periodontal ligament cell viability for avulsed permanent teeth prior to their replantation. The solutions used for comparison with HBSS were saline, milk, Ocimum sanctum extract, Morinda citrifolia juice, Ringer’s lactate, egg albumin, coconut milk, platelet-rich fibrin, coconut water, propolis, pomegranate juice, Placentrex, and aloe vera.

The meta-analysis presented results that consistently showed HBSS to be the solution that preserved the highest number of viable PDL cells compared to the other tested storage media. The SMD for HBSS compared to other media was 2.50 (95% CI: 0.85–4.15), *p* = 0.003, validating a statistically significant advantage in HBSS being able to maintain viable PDL cells. When HBSS was compared to aloe vera, the results showed an SMD of 4.48 (95% CI: 0.92–8.03), *p* = 0.014, again reinforcing the superiority of HBSS as a storage medium. However, while HBSS proved to be the most successful medium, alternative solutions demonstrated promising results in various investigations. For example, propolis, coconut water, and Morinda citrifolia juice reported a higher number of viable PDL cells, suggesting their ability to serve as potential substitutes in emergency situations when HBSS is unavailable. Nonetheless, the overall findings supported HBSS as the gold standard in the preservation of PDL cell viability.

The strengths of this study lie in its rigorous methodology and comprehensive approach in analyzing the effectiveness of HBSS in preserving PDL cell viability. The systematic review adheres to the PRISMA guidelines, ensuring transparency and reproducibility in the selection and evaluation of the chosen studies. In addition, the inclusion of the meta-analysis enriches the statistical power of such findings by the synthesis of data from multiple sources, allowing for a more reliable comparison of HBSS with other storage media. Moreover, the use of multiple electronic databases minimized the risk of selection bias, thus ensuring a broad and diverse data set. Furthermore, the study encompasses standardized eligibility criteria and robust statistical methods, which account for the variability found amongst the studies. Therefore, these factors collectively contribute to the strength and credibility of the study’s conclusion, reinforcing HBSS as the most effective storage medium in the preservation of PDL cell viability in avulsed permanent teeth.

However, on the other hand, there are several limitations that must be taken into account when interpreting the findings. Firstly, a major limitation found in the analysis was the limited number of studies included, which led to a small sample size resulting in a lower generalizability of the results, implying the need for future clinical studies to have larger datasets to strengthen the evidence that HBSS is the optimal storage medium following avulsion. Another important factor that was found was the variation in the storage times. The studies stored the teeth for periods ranging from 15 min up to 120 min. Yet, in reality, when these accidents occur, patients usually seek dental care within at least two hours from the time of the accident. As such, delays in seeking emergency care extend the extra-oral dry time of the tooth, which is a critical factor that affects the viability of the PDL cells. Therefore, future research should focus on longer storage times that reflect real-life scenarios. Additionally, in order to carry out the study, the teeth that were used in the in vitro study were extracted, mainly due to orthodontic reasons and then the investigation was performed in vitro. In vitro studies do not replicate real-life conditions, which in this case can impact the clinical applicability of the findings. When avulsion occurs, in most circumstances, the tooth is contaminated by bacteria or debris due to contact with the ground/surface where the accident occurs. As such, when the teeth were extracted, they did not undergo or experience the effects of real-life variables such as those faced during dental avulsion (saliva composition, bacterial contamination, temperature fluctuations, mechanical forces, etc.). Moreover, in vitro studies conducted on extracted teeth do not incorporate true physiological conditions. For example, factors such as blood supply, immune response, and healing mechanisms play a crucial role in PDL cell survival and regeneration following replantation. In addition to this, they also lack systemic influences of the body’s natural response to trauma, such as inflammation, immune cell activity, and revascularization. Furthermore, the variability in the handling of the teeth can also raise concerns in in vitro studies. The differences in how the teeth were extracted and handled can introduce inconsistencies between the studies, making it difficult to standardize the results. For example, the influence of extraction-related stress caused by the forces exerted upon the tooth during the extraction varies not only in the type of tooth extracted but also by the operator performing the extraction. This means that the level of trauma sustained by the PDL during the extraction would have fluctuated across the different studies as the extractions were performed by different operators and would not have accurately recorded the exact forces sustained in a real-life avulsion case. As such, this variability could have contributed to the high heterogeneity observed in the analysis. Furthermore, although the majority of the in vitro studies used in this review were conducted on premolars, one of the articles by Shetty (2019) [19] conducted their study on molars. The in vitro storage of these particular types of teeth presents another limitation, as the maxillary incisors are the more commonly affected teeth by avulsion due to their position in the arch. Thus, again, in vitro studies fail to replicate real-life situations. Also, premolars have a larger root surface area compared to the incisors, meaning they naturally contain a higher number of PDL cells. Thus, the chosen teeth used in the in vitro studies, along with the differences in the PDL cell density, influence the outcomes, making it challenging to directly apply the results to the incisors. The majority of replanted teeth eventually present with root resorption over time as the frequency of periodontal healing is around 20% [1]. Because in vitro studies only conduct short-term observations, assessing PDL cell viability within several minutes or hours, these types of studies fail to provide insights into long-term healing, reattachment, or the risks that can arise following replantation, such as ankylosis or resorption [25]. Moreover, in vitro studies present a challenge in translating the results to clinical outcomes because even if a storage medium preserves a high percentage of viable PDL cells in vitro, it is not a guarantee for successful replantation or reattachment in a patient, as factors such as the condition of the alveolar socket, splinting technique used, and post-replantation care play significant roles.

As such, due to these limitations found in in vitro studies, future research should incorporate these considerations and ensure that environmental factors and true physiological conditions are accurately replicated and accounted for. In addition to this, the evaluation of the success of the storage media in terms of clinical outcomes would provide a more comprehensive understanding of the actual effectiveness of the media. This could include the investigation into tooth survival, long-term periodontal health, and functional reattachment following replantation. Likewise, it would be interesting in future studies to incorporate clinical follow-ups following the reimplantation of avulsed teeth stored in different media to assess which is the most effective for long-term tooth retention and revascularization. Without these long-term follow-up studies, it remains unclear whether storage media that maintain high PDL cell viability in vitro ultimately lead to better clinical outcomes. Expanding on this research with additional long-term studies could strengthen and enhance the clinical relevance of these findings and help refine the guidelines for the optimal and effective management of avulsed teeth.

Moreover, there are numerous other solutions, other than the ones studied in this investigation, that can be compared to HBSS in order to ascertain more practical and realistic recommendations for emergency situations where HBSS is usually not readily available. Solutions such as cornisol have shown promising results in ex vivo studies. It has the potential to maintain the viability of the PDL cells due to the presence of sodium bicarbonate that acts as a buffer and maintains a suitable pH and osmolarity of 7.4 and 290 mOsm. A key component found in Cornisol, chondroitin sulfate, maintains the cell membrane due to its antioxidant properties and its ability to maintain physiological pH despite changes in carbon dioxide concentration produced by cellular respiration, which could be a potential reason behind the higher cell viability found in Cornisol solutions [26]. Moreover, rice water has been suggested as another type of medium for the storage of avulsed teeth. In certain studies, it has been shown to maintain the highest number of viable cells, which can be attributed to its low sodium content, useful quantities of potassium, vitamin B, thiamine and niacin, along with its anti-inflammatory properties [27]. Research has also proposed the use of honey as a type of storage medium due to the rich nutrient composition and high levels of antioxidants it contains. Its antioxidant properties are able to remove the free radicals produced by the inflammatory response due to avulsion, allowing protection against lipid peroxidation of the cell membrane, thus enabling cell proliferation via the stimulation of epithelial cells, which play an essential role in the healing process and repair of the damaged tissue [28]. Similarly, investigations into the use of green tea and turmeric have been conducted. Green tea, an increasingly popular herbal drink, has shown enthusiastic results in research with the ability to maintain 90% of PDL cell viability for up to 24 h. However, turmeric extract, although it did not show as promising results, was still able to obtain 81.63% of viable cells following storage [29]. As these solutions were not able to be discussed in depth in this particular investigation, future research could explore their potential use for maintaining periodontal cell viability in comparison to HBSS following the previously mentioned recommendations.

Nonetheless, based on the results found in this systematic review and meta-analysis, the optimal choice of storage medium in emergency situations of dental avulsion, HBSS, remains the most effective and reliable medium for PDL cell preservation. However, as mentioned before, when HBSS is not available, coconut water would be the next most accessible solution to most people. Although Morinda citrifolia juice showed promising results exceeding those of HBSS, it is a solution native to Australia and Southeast Asia; thus, its accessibility in other countries could be limited. If these solutions cannot be obtained for certain reasons, milk is another recommendation, preferably milk with a low fat content, which can be accessed with relative ease in most places nowadays. It contains essential nutrients to support cell health, and although it is not as effective as HBSS, it still provides a good short-term solution. Likewise, aloe vera is another less optimal solution but is still able to provide some degree of preservation; thus, its total exclusion as a storage medium altogether is not recommended. Despite outlining these recommendations, it all comes down to where the avulsion occurs and the availability of solutions in the area. While immediate reimplantation is the best course of action, it cannot always be performed; thus, clinical professionals should prioritize HBSS when available and consider these alternative options when necessary.

## 6. Conclusions

The information and results provided in this systematic review and meta-analysis demonstrated sufficient evidence concluding that HBSS is the most effective storage medium for preserving the greatest amount of viable periodontal ligament cells following the avulsion of permanent teeth. The meta-analysis revealed that teeth stored in HBSS exhibited considerably higher PDL cell viability compared to other storage media, with a strong positive effect size and high statistical significance. Nonetheless, among the other solutions that were evaluated alongside HBSS, Morinda citrifolia juice, propolis, and coconut water emerged with results comparable to those of HBSS, suggesting their use as promising alternative storage mediums. The formulation of both a systematic review and meta-analysis strengthened the reliability of the findings and encompassed a comprehensive literature search across three major databases to ensure a robust data collection. In addition to this, the use of the modified CONSORT checklist and PRISMA statement ensured quality evaluation and minimized bias across the work. However, the study did include various limitations. The high heterogeneity that was found amongst the studies was apparent but can be accounted for due to the difference in the sample sizes (i.e., differences in the number of teeth in which the experiment was conducted, which could have affected the generalizability of the results), the type of extracted teeth (i.e., some studies used premolars and others molars), and the experimental methods used. Additionally, another prominent limitation is the limited availability of accessing HBSS, especially when emergency situations arise. Thus, this can limit its practical application in situations when avulsion does occur. Because of this, further research should look into exploring the accessibility and practicality of HBSS, especially in locations where traumatic dental injuries are more likely to occur (i.e., schools/sports facilities). Moreover, Supplementary Materials research should be performed to investigate solutions such as Ocimum sanctum extract, propolis and coconut water as alternative media due to their promising results. Lastly, standardized methodologies across future studies would help to reduce variability and provide more reliable data for clinical guidelines.

## Figures and Tables

**Table 1:** Inclusion criteria.

Category	Inclusion Criteria
Type of study	Publications in English published between 2014 and 2024; in vitro studies; studies on humans; studies on permanent teeth.
Type of patient/teeth	Extracted human teeth that are non-carious, have healthy periodontium without severe periodontal disease, and have intact roots
Type of intervention	Storage media that included HBSS as one of the solutions measured
Type of outcome variables	Studies providing data on the number of viable periodontal ligament cells present after the storage of the avulsed teeth in different storage media

**Table 2:** Exclusion criteria.

Category	Exclusion Criteria
Type of study	Systematic reviews; case reports; case studies; abstracts; meta-analyses; studies on animals
Type of patient/teeth	Teeth that are carious, have severe periodontal disease, or have fractured or non-intact roots
Type of intervention	Studies that do not include HBSS as one of the storage media used to assess PDL cell viability
Type of outcome variable	Data from studies conducted in vivo or studies that measure non-viable PDL cells, as the aim of the meta-analysis is to obtain quantitative data on viable PDL cells

**Table 3:** MeSH terms and Boolean operators used in each database.

Database	Keywords
EBSCO (PubMed-Medline)	((((avulsed permanent teeth) AND (periodontal cell viability)) AND (hanks balanced salt solution)) OR (storage media)) AND (in vitro)
Web of Science	((((avulsed permanent teeth) AND (periodontal cell viability)) AND (hanks balanced salt solution)) OR (storage media)) AND (in vitro)
Scopus	((((avulsed permanent teeth) AND (periodontal cell viability)) AND (hanks balanced salt solution)) OR (storage media)) AND (in vitro)

**Table 4:** Results table of the data extracted from the final selected studies.

Article	Type of Study	Author/s	Year	Sample Size	Types of Storage Media	Complications	Best Storage Media Based on n° of PDL Cells
An In Vitro Evaluation of Morinda citrifolia and Ocimum sanctum as Potential Storage Media to Maintain Cell Viability for Avulsed Teeth Using Collagenase Dispase Assay	In Vitro Study	Shweta Vijaykumar Sagare, Anad Patil, Pranav Patil, R. Susheel Kumar, Sairam Gangishetti, Priya Ingale [16]	2023	45 teeth	HBSS. Ocimum sanctum extract. Morinda citrifolia juice.	N/A	In order from most viable cells to the least: Morinda Citrifolia juice. HBSS. Ocimum sanctum extract. Morinda Citrifolia juice has potential as a storage medium and as an alternative to HBSS.
Assessment of the Efficacy of Different Storage Media for Maintaining an Avulsed Tooth	In Vitro Study	AlWaleed Abushana, Abdulfatah Alazmah, Uthman S Uthman, Adel S Alqarni, Abdulhamid Al Ghwainem, Narendra Varma Penumatsa [18]	2022	80 teeth	HBSS. Ringer’s lactate. Aloe vera. Egg albumin.	N/A	In order from most viable cells to the least: HBSS. Aloe vera. Egg albumin. Ringer’s lactate. HBSS exhibited the highest efficacy as a storage medium for an avulsed tooth.As an alternative, aloe vera could be used when HBSS is not available.
Coconut milk and probiotic milk as storage media to maintain periodontal ligament cell viability	In Vitro Study	Divya Saini, Prahlad Gadicherla, Prakash Chandra, Latha Anandakrishna [10]	2017	69 teeth	HBSS. Coconut milk. Probiotic milk.	N/A	In order from most viable cells to the least: HBSS. Probiotic milk. Coconut milk. Probiotic milk was able to maintain PDL cell viability as well as HBSS, but coconut milk may not be suitable as an interim transport medium.
Comparative evaluation of the efficacy of platelet-rich fibrin and Hank’s balanced salt solution as a storage medium for avulsed teeth	In vitro study	Ashwija Shetty, Somnath Ghosh, A Srirekha, T Jaykumar, Champa Chikkamallaiah, Savitha Adiga [19]	2019	20 teeth	HBSS. Platelet-rich fibrin.		In order from most viable cells to the least: Platelet rich fibrin. HBSS.
Comparative evaluation of the efficacy of aloe vera gel with milk and Hank’s balanced salt solution in maintaining the viability of the PDL cells in avulsed teeth		Baren Abraham, Parvathy Kumaran, B R Varma, Arun Mamachan Xavier, Suresh J Kumar [20]	2019	30 teeth	HBSS. Aloe vera gel. Low-fat cow’s milk.	N/A	In order from most viable cells to the least: HBSS. Milk. Aloe vera. Milk can be used as an alternative to HBSS for PDL cell viability.
Efficacy of natural coconut water, pre-packaged coconut water, and Hank’s balanced salt solution as storage media in maintaining periodontal ligament cell viability	In Vitro Study	Sara Samreen, Rituraj Kesri, Ankita Ukey, Pratik Surana, Anshuta Sahu, Pankaj Agrawal, Owais Rahman [21]	2024	24 teeth	HBSS. Natural coconut water. Pre-packaged coconut water.	N/A	In order from most viable cells to the least: Natural coconut water. HBSS. Pre-packaged coconut water.
In vitro comparative evaluation of different storage media (Hank’s balanced salt solution, propolis, aloe vera, and pomegranate juice) for preservation of avulsed teeth	In Vitro Study	Prashant Babaji, Mahesh Melkundi, Raghu Devanna, Suresh B.S., Vishwajit Rampratap Chaurasia, Gopinath P.V. [22]	2017	40 teeth	HBSS. Propolis. Aloe vera. Pomegranate juice.	N/A	In order from most viable cells to the least: Propolis. HBSS. Aloe vera. Pomegranate juice.
Nature’s benefaction as a lifesaver for an avulsed tooth	In Vitro Study	Saumya Navit, Niharika Shahi, Suleman Abbas Khan, Anshul Sharma, Vartika Singh, Ratna Priya Mishra, Pragati Navit, Prerna Sharma [23]	2017	48 teeth	HBSS. Coconut water. Aloe vera. Saline.	N/A	In order from most viable cells to the least: HBSS. Coconut water. Aloe vera. Saline. HBSS is the most effective storage medium in maintaining PDL cell viability.
Comparative analysis of the effectiveness of four different storage media (Placentrex, propolis 10%, pomegranate juice 5%, and Hank’s balanced salt solution) in preserving the viability of periodontal ligament cells	In Vitro Study	Musaffar Thoyalil, Dhanya Kamalakshan Belchada, Konsam Bidya Devi, Rekha Vasantha Ravi, Mridhul Madathikandy Uchummal, Ramnesh Parikkal [24]	2023	60 teeth	HBSS. Placentrex. Propolis 10%. Pomegranate juice 5%.	N/A	In order from most viable cells to the least: HBSS. Placentrex. Pomegranate juice. Propolis. All the other storage media studied were significantly inferior to HBSS.

**Table 5:** Final input of data for model 1 meta-analysis [10,16,18,19,20,21,22,23,24].

	TX			Control		
AUTHOR	nTX	mTX	sTX	nCT	mCT	sCT
Sagare et al., 2023 [16]	15	84.30	12.50	30	76.61	13.92
Abushana et al., 2022 [18]	20	38.48	2.32	60	25.35	2.43
Saini et al., 2017 [10]	23	144.79	14.40	46	76.00	12.39
Shetty et al., 2019 [19]	10	76,800.00	4727.15	10	79,072.00	7570.25
Abraham et al., 2019 [20]	10	921.40	608.44	20	526.85	321.87
Samreen et al., 2024 [21]	8	79.88	2.69	16	79.94	5.64
Babaji et al., 2017 [22]	10	262.00	3.13	30	241.67	3.65
Navit et al., 2017 [23]	12	87.33	5.24	36	67.01	4.39
Thoyalil et al., 2023 [24]	15	73.12	7.41	45	60.96	11.47

n = number of teeth; m = mean; s = SD; TX = test HBSS group; CT = other groups.

**Table 6:** Model 1 results of the meta-analysis of standardized mean differences in PDL counts: mean difference (SMD) HBSS-Other, standard error (SE), 95% confidence interval, z test (*p*-value), I^2^ index, and Cochran’s Q statistic (*p*-value) for heterogeneity; Egger’s test (*p*-value) for publication bias.

SMD	SE	95% CI	z (*p*-Value)	I^2^	Q_H_ (*p*-Value)	Egger (*p*-Value)
2.50	0.84	0.85, 4.15	0.003 **	97.0%	<0.001 ***	0.004 **

** *p* < 0.01; *** *p* < 0.001.

**Table 7:** Final input of data for model 2 meta-analysis.

	TX			Control		
AUTHOR	nTX	mTX	sTX	nCT	mCT	sCT
Abushana et al., 2022 [15]	20	38.48	2.32	20	30.36	1.86
Abraham et al., 2019 [20]	10	921.40	608.44	10	241.00	194.57
Babaji et al., 2017 [22]	10	262.00	3.13	10	226.00	3.43
Navit et al., 2017 [23]	12	87.33	5.24	12	70.59	4.73

n = number of teeth; m = mean; s = SD; TX = Test HBSS group; CT = Other groups.

**Table 8:** Model 2 results of the meta-analysis of standardized mean differences in PDL counts: mean difference (SMD) HBSS-Aloe, standard error (SE), 95% confidence interval, z test (*p*-value), I^2^ index, and Cochran’s Q statistic (*p*-value) for heterogeneity; Egger’s test (*p*-value) for publication bias.

SMD	SE	95% CI	z (*p*-Value)	I^2^	Q_H_ (*p*-Value)	Egger (*p*-Value)
4.48	1.81	0.92, 8.03	0.014 *	96.7%	<0.001 ***	<0.001 ***

* *p* < 0.05; *** *p* < 0.001.

## Data Availability

The data presented in this study are available on request from the corresponding author.

## Supplementary Materials

The following are available online at https://www.mdpi.com/article/10.3390/jcm14061986/s1. Table S1. PRISMA Statement for included studies, Table S2. Modified CONSORT Checklist for in vitro studies, Table S3. (results table of the data extracted from the final selected studies), meta-analysis results Tables S4–S7 and Figures S1–S7, Tables on the search results (S8), and exclusion reasons in the process of assessing the reports for eligibility (S8).
