# Supplementary material for: The Comparison Between the Different Types of Storage Mediums on the Viability of Periodontal Cells Prior to the Replantation of Avulsed Teeth: A Systematic Review & Meta-Analysis
Source: J Clin Med. 2025 Mar 14;14(6):1986. doi: 10.3390/jcm14061986 (PMC11942730; doi:10.3390/jcm14061986)
Supplement: Supplementary file 1 [file jcm-14-01986-s001.zip › jcm-3499371-supplementary.pdf]

PRISMA Statement

The results of the PRISMA checklist for the 9 articles included within this study were considered to be low (shown in table 11). The included articles were in vitro studies rather than systematic reviews or meta-analyses, thus the PRSIMA Statement was not fully applicable, as in vitro studies do not follow systematic review protocols. Furthermore, many of the PRISMA checklist items do not directly apply to primary laboratory research included within the selected studied.

Table S1: PRISMA Statement for included studies

	**Checklist item**	**Article**								
		Sagare et al. 2023	Abushana et al. 2022	Saini et al. 2017	Shetty et al. 2019	Abraham et al. 2019	Samreen et al. 2024	Babaji et al. 2017	Navit et al. 2017	Thoyalil et al. 2023
**TITLE**										
Title	Identify the report as a systematic review	NO	NO	NO	NO	NO	NO	NO	NO	NO
**ABSTRACT**										
Abstract	See the PRISMA 2020 for Abstract checklist	YES, page 1	YES, page 1	YES, page 1	YES, page 1	YES, page 1	YES, page 1	YES, page 1	YES, page 1	YES, page 1
**INTRODUCTION**										
Rationale	Describe the rationale for the review in the context of existing knowledge.	YES, page 1	YES, page 2	YES, page 2	YES, page 2	YES, page 1	YES, page 1	YES, page 2	YES, page 1	YES, page 1
Objectives	Provide an explicit statement of the objective(s) or question(s) the review addresses.	YES, page 2	YES, page 1	YES, page 1	YES, page 1	YES, page 1	YES, page 1	YES, page 1	YES, page 1	YES, page 1
**METHODS**										

Eligibility criteria	Specify the inclusion and exclusion criteria for the review and how studies were grouped for the syntheses.	YES, page 3	YES, page 2	YES, page 2	YES, page 2	YES, page 2	YES, page 2	YES, page 2	YES, page 2	YES, page 2
Information sources	Specify all databases, registers, websites, organisations, reference lists and other sources searched or consulted to identify studies. Specify the date when each source was last searched or consulted.	NO	NO	NO	NO	NO	NO	NO	NO	NO
Search strategy	Present the full search strategies for all databases, registers and websites, including any filters and limits used.	NO	NO	NO	NO	NO	NO	NO	NO	NO
Selection process	Specify the methods used to decide whether a study met the inclusion criteria of the review, including how many reviewers screened each record and each report retrieved, whether they worked independently, and if applicable, details of automation tools used in the process.	NO	NO	NO	NO	NO	NO	NO	NO	NO
Data collection process	Specify the methods used to collect data from reports, including how many reviewers collected data from each report, whether they	YES, page 4	YES, page 4	YES, page 4	YES, page 3	YES, page 2	YES, page 2	YES, page 2	YES, page 2	YES, page 2

	worked independently, any processes for obtaining or confirming data from study investigators, and if applicable, details of automation tools used in the process.									
Data items	List and define all outcomes for which data were sought. Specify whether all results that were compatible with each outcome domain	YES, page 4	YES, page 4	YES, page 4	YES, page 3	YES, page 2	YES, page 2	YES, page 2	YES, page 2	YES, page 2
	In each study were sought (e.g. for all measures, time points, analyses), and if not, the methods used to decide which results to collect.	YES, page 4	YES, page 4	YES, page 4	YES, page 3	YES, page 2	YES, page 3	YES, page 2	YES, page 2	YES, page 2
	List and define all other variables for which data were sought (e.g. participant and intervention characteristics, funding sources).<br>Describe any assumptions made about any missing or unclear information.	YES, page 4	YES, page 4	YES, page 4	YES, page 3	YES, page 2	YES, page 2	YES, page 2	YES, page 2	YES, page 2
Study risk of bias assessment	Specify the methods used to assess risk of bias in the included studies, including details of the tool(s) used, how many reviewers assessed each study and whether they worked independently, and if applicable, details of	NO	NO	NO	NO	NO	NO	NO	NO	NO

	automation tools used in the process.									
Effect measures	Specify for each outcome the effect measure(s) (e.g. risk ratio, mean difference) used in the synthesis or presentation of results.	YES, page 5	YES, page 3	YES, page 3	YES, page 3	YES, page 2	YES, page 3	YES, page 3	YES, page 2	YES, page 7
Synthesis methods	Describe the processes used to decide which studies were eligible for each synthesis (e.g. tabulating the study intervention characteristics and comparing against the planned groups for each synthesis (item #5)).	YES, page 5	YES, page 3	YES, page 3	YES, page 3	YES, page 2	YES, page 3	YES, page 3	YES, page 2	YES, page 7
	Describe any methods required to prepare the data for presentation or synthesis, such as handling of missing summary statistics, or data conversions.	NO	NO	NO	NO	NO	NO	NO	NO	NO
	Describe any methods used to tabulate or visually display results of individual studies and syntheses.	NO	NO	NO	NO	NO	NO	NO	NO	NO
	Describe any methods used to synthesize results and provide a rationale for the choice(s). If meta-analysis was performed, describe the model(s), method(s) to identify the presence and extent of statistical heterogeneity, and	NO	NO	NO	NO	NO	NO	NO	NO	NO

	software package(s) used.									
	Describe any methods used to explore possible causes of heterogeneity among study results (e.g. subgroup analysis, meta- regression).	NO	NO	NO	NO	NO	NO	NO	NO	NO
	Describe any sensitivity analyses conducted to assess robustness of the synthesized results.	NO	NO	NO	NO	NO	NO	NO	NO	NO
Reporting bias assessment	Describe any methods used to assess risk of bias due to missing results in a synthesis (arising from reporting biases).	NO	NO	NO	NO	NO	NO	NO	NO	NO
Certainty assessment	Describe any methods used to assess certainty (or confidence) in the body of evidence for an outcome.	NO	NO	NO	NO	NO	NO	NO	NO	NO
**RESULTS**										
Study selection	Describe the results of the search and selection process, from the number of records identified in the search to the number of studies included in the review, ideally using a flow diagram.	NO	NO	NO	NO	NO	NO	NO	NO	NO
	Cite studies that might appear to meet the inclusion criteria, but which were excluded and explain why they were excluded.	NO	NO	NO	NO	NO	NO	NO	NO	NO

Study characteristics	Cite each included study and present its characteristics.	YES, page 5	YES, page 4	YES, page 3	YES, page 3	YES, page 3	YES, page 3	YES, page 3	YES, page 3	YES, page 11
Risk of bias in studies	Present assessments of risk of bias for each included study.	NO	NO	NO	NO	NO	NO	NO	NO	NO
Results of individual studies	For all outcomes, present, for each study: (a) summary statistics for each group (where appropriate) and (b) an effect estimate and its precision (e.g. confidence/credible interval), ideally using structured tables or plots.	YES, page 6	YES, page 4	YES, page 3	YES, page 3	YES, page 3	YES, page 3	YES, page 3	YES, page 3	YES, page 11
Results of syntheses	For each synthesis, briefly summarise the characteristics and risk of bias among contributing studies.	NO	NO	NO	NO	NO	NO	NO	NO	NO
	Present results of all statistical syntheses conducted. If meta- analysis was done, present for each the summary estimate and its precision (e.g. confidence/credible interval) and measures of statistical heterogeneity. If comparing groups, describe the direction of the effect.	NO	NO	NO	NO	NO	NO	NO	NO	NO
	Present results of all investigations of possible causes of heterogeneity among study results.	NO	NO	NO	NO	NO	NO	NO	NO	NO

	Present results of all sensitivity analyses conducted to assess the robustness of the synthesized results.	NO	NO	NO	NO	NO	NO	NO	NO	NO
Reporting biases	Present assessments of risk of bias due to missing results (arising from reporting biases) for each synthesis assessed.	NO	NO	NO	NO	NO	NO	NO	NO	NO
Certainty of evidence	Present assessments of certainty (or confidence) in the body of evidence for each outcome assessed.	NO	NO	NO	NO	NO	NO	NO	NO	NO
**DISCUSSION**										
Certainty of evidence	Provide a general interpretation of the results in the context of other evidence.	YES, page 6	YES, page 4	YES, page 4	YES, page 3	YES, page 3	YES, page 3	YES, page 3	YES, page 3	YES, page 9
	Discuss any limitations of the evidence included in the review.	YES, page 6	YES, page 4	YES, page 4	YES, page 4	YES, page 4	YES, page 4	NO	YES, page 3	YES, page 11
	Discuss any limitations of the review processes used.	NO	NO	NO	NO	NO	NO	NO	NO	NO
	Discuss implications of the results for practice, policy, and future research.	YES, page 6	YES, page 4	YES, page 4	YES, page 4	YES, page 4	YES, page 4	YES, page 4	YES, page 3	YES, page 11
**OTHER INFORMATION**										
Registration and protocol	Provide registration information for the review, including register name and registration number, or state that the review was not registered.	NO	NO	NO	NO	NO	NO	NO	NO	NO

	Indicate where the review protocol can be accessed, or state that a protocol was not prepared.	NO	NO	NO	NO	NO	NO	NO	NO	NO
	Describe and explain any amendments to information provided at registration or in the protocol.	NO	NO	NO	NO	NO	NO	NO	NO	NO
Support	Describe sources of financial or non-financial support for the review, and the role of the funders or sponsors in the review.	YES, page 7	NO	NO	YES, page 4	YES, page 5	YES, page 4	YES, page 4	YES, page 4	YES, page 11
Competing interests	Declare any competing interests of review authors.	YES, page 7	NO	YES, page 5	YES, page 4	YES, page 5	YES, page 4	YES, page 4	YES, page 4	YES, page 11
Availability of data, code and other materials	Report which of the following are publicly available and where they can be found template data collection forms; data extracted from included studies; data used for all analyses; analytic code; any other materials used in the review.	NO	NO	NO	NO	NO	NO	NO	NO	NO
**TOTAL**		17/42	15/42	16/42	17/42	17/42	17/42	16/42	17/42	17/42

Modified CONSORT Checklist for In-Vitro Studies

The results following the evaluation of risk of bias across the in vitro studies using the modified CONSORT checklist can be seen in Table 10 below. The quality and risk of bias of each study was evaluated using various items consisting of the abstract, background, objectives and/or hypotheses, intervention, implementation, statistical methods, outcome and estimations, limitations and trial protocols (38). Following the completion of the checklist, it was observed that only one out of the nine studies did not mention their limitations and another two studies did not include any funding information. There was also no mention of randomised sequence generation, allocation concealment method, blinding, implementation, nor where the full trial protocol can be accessed in any of the nine studies. Despite this, in all of the 9 studies the abstract, background and objectives, interventions, sample sizes, statistical methods, outcomes and estimation were provided. The overall results of risk of bias was concluded to be low in each study.

Table S2: Modified CONSORT Checklist for in vitro studies

**Modified CONSORT checklist used to assess quality and risk of bias of in vitro studies**										
**Section/Topic**	**Checklist item**	Sagare et al. 2023	Abushana et al. 2022	Saini et al. 2017	Shetty et al. 2019	Abraham et al. 2019	Samreen et al. 2024	Babaji et al. 2017	Navit et al. 2017	Thoyalil et al. 2023
Abstract	Item 1. Structured summary of trial design, methods, results, and conclusions	YES: page 1	YES: page 1	YES: page 1	YES: page 1	YES: page 1	YES: page 1	YES: page 1	YES: page 1	YES: page 1
**Introduction**										
Background and objectives	Item 2a. Scientific background and explanation of rationale<br>Item 2b. Specific objectives and/or	YES: pages 1, 2	YES: page 2	YES: page 2	YES: page 2	YES: page 1	YES: page 1	YES: page 2	YES: page 1	YES: page 2

	hypotheses									
**Methods**										
Intervention	Item 3. The intervention for each group, including how and when it was administered, with sufficient detail to enable replication	YES: pages 2, 3	YES: pages 2, 3	YES: page 2	YES: page 2	YES: page 2	YES: page 2	YES: pages 2, 3	YES: pages 1, 2	YES: pages 2, 3
Outcomes	Item 4. Completely defined, pre-specified primary and secondary measures of outcome, including how and when they were assessed	YES: page 6	YES: pages 2, 3	YES: page 2	YES: page 2	YES: page 2	YES: page 2	YES: page 3	YES: page 2	YES: pages 2, 3
Sample size	Item 5. How sample size was determined	YES: page 2	YES: page 2	YES: page 2	YES: page 2	YES: page 1, 2	YES: page 2	YES: page 2	YES: page 2	YES: page 2
Randomization Sequence generation	Item 6. Method used to generate the random allocation sequence	NO	NO	NO	NO	NO	NO	NO	NO	NO
Allocation concealment mechanism	Item 7. Mechanism used to implement the random allocation sequence	NO	NO	NO	NO	NO	NO	NO	NO	NO

	(for example, sequentially numbered containers), describing any steps taken to conceal the sequence until intervention was assigned									
Implementation	Item 8. Who generated the random allocation sequence, who enrolled teeth,	NO	NO	NO	NO	NO	NO	NO	NO	NO
Blinding	Item 9. If done, who was blinded after assignment to intervention (for example, care providers, those assessing outcomes), and how	NO	NO	NO	NO	NO	NO	NO	NO	NO
Statistical methods	Item 10. Statistical methods used to compare groups for primary and secondary outcomes	YES: page 3	YES: page 3	YES: page 2	YES: page 2	YES: page 2	YES: page 2	YES: page 3	YES: page 2	YES: page 5
**Results**										
Outcomes and estimation	Item 11. For each primary	YES: page 4	YES: page 3	YES: pages 2, 3	YES: pages 2, 3	YES: page 2	YES: pages 2, 3	YES: page 3	YES: pages 2, 3	YES: pages 5, 8

	and secondary outcome, results for each group,									
**Discussion**										
Limitations	Item 12. Trial limitations, addressing sources of potential bias, imprecision,	YES: page 6	YES: page 4	YES: page 4	YES: page 4	YES: page 4	YES: page 4	NO	YES: page 3	YES: page 11
**Other Information**										
Funding	Item 13. Sources of funding and other support (for example suppliers of drugs), role of funders	YES: page 7	NO	NO	YES: page 4	YES: page 5	YES: page 4	YES: page 4	YES: page 4	YES: page 11
Protocol	Item 14. Where the full trial protocol can be accessed, if available	NO	NO	NO	NO	NO	NO	NO	NO	NO
**Overall risk of bias**										
		Low risk	Low risk	Low risk	Low risk	Low risk	Low risk	Low risk	Low risk	Low risk

Table S3: Results table of the data extracted from the final selected studies

Article	Type of study	Author/s	Year	Sample size	Types of storage media	Compli-cations	Best storage media based on nº of PDL cells
An In Vitro Evaluation of Morinda citrifolia and Ocimum sanctum as Potential Storage Media to Maintain Cell Viability for Avulsed Teeth Using Collagenase Dispase Assay	In Vitro Study	Shweta Vijaykumar Sagare, Anad Patil, Pranav Patil, R. Susheel Kumar, Sairam Gangishetti, Priya Ingale	2023	45 teeth	1. HBSS<br>2. Ocimum sanctum extract<br>3. Morinda citrifolia juice	N/A	In order from most viable cells to the least<br>1. Morinda Citrifolia juice<br>2. HBSS<br>3. Ocimum sanctum extract<br><br>Morinda Citrifolia juice has potential as a storage medium and as an alternative to HBSS
Assessment of the Efficacy of Different Storage Media for Maintaining an Avulsed Tooth	In Vitro Study	AlWaleed Abushana, Abdulfatah Alazmah, Uthman S Uthman, Adel S Alqarni, Abdulhamid Al Ghwainem, Narendra Varma Penumatsa	2022	80 teeth	1. HBSS<br>2. Ringer´s lactate<br>3. Aloe vera<br>4. Egg albumin	N/A	In order from most viable cells to the least<br>1. HBSS<br>2. Aloe vera<br>3. Egg albumin<br>4. Ringer´s lactate<br><br>HBSS exhibited the highest efficacy as a storage medium for an avulsed tooth.<br>as an alternative aloe vera could be used when HBSS is not available

Coconut milk and probiotic milk as storage media to maintain periodontal ligament cell viability	In Vitro Study	Divya Saini, Prahlad Gadicherla, Prakash Chandra, Latha Anandakrishna	2017	69 teeth	1. HBSS<br>2. Coconut milk<br>3. Probiotic milk	N/A	In order from most viable cells to the least<br>1. HBSS<br>2. Probiotic milk<br>3. Coconut milk<br><br>Probiotic milk was able to maintain PDL cell viability as well as HBSS, but coconut milk may not be suitable as an interim transport media
Comparative evaluation of efficacy of platelet-rich fibrin and Hank´s balanced salt solution as a storage medium for avulsed teeth	In-vitro study	Ashwija Shetty, Somnath Ghosh, A Srirekha, T Jaykumar, Champa Chikkamallaiah, Savitha Adiga	2019	20 teeth	1. HBSS<br>2. Platelet-rich fibrin		In order from most viable cells to the least<br>1. Platelet rich fibrin<br>2. HBSS
Comparative evaluation of the efficacy of aloe vera gel with milk and hanks balanced salt solution in maintaining the viability of the PDL cells in Avulsed teeth		Baren Abraham, Parvathy Kumaran, B R Varma, Arun Mamachan Xavier, Suresh J Kumar	2019	30 teeth	1. HBSS<br>2. Aloe vera gel<br>3. Low-fat cow´s milk	N/A	In order from most viable cells to the least<br>1. HBSS<br>2. Milk<br>3. Aloe vera<br><br>Milk can be used as an alternative to HBSS for PDL cell viability

Efficacy of Natural coconut water, pre-packaged coconut water, and Hanks balanced salt solution as storage media in maintaining periodontal ligament cell viability	In Vitro Study	Sara Samreen, Rituraj Kesri, Ankita Ukey, Pratik Surana, Anshuta Sahu, Pankaj Agrawal, Owais Rahman	2024	24 teeth	1. HBSS<br>2. Natural coconut water<br>3. Pre-packaged coconut water	N/A	In order from most viable cells to the least<br>1. Natural coconut water<br>2. HBSS<br>3. Pre-packaged coconut water
In vitro comparative evaluation of different storage media (Hanks balanced salt solution, propolis, aloe vera, and pomegranate juice) for preservation of avulsed tooth	In Vitro Study	Prashant Babaji, Mahesh Melkundi, Raghu Devanna, Suresh B.S., Vishwajit Rampratap Chaurasia, Gopinath P.V.	2017	40 teeth	1. HBSS<br>2. Propolis<br>3. Aloe vera<br>4. Pomegranate juice	N/A	In order from most viable cells to the least<br>1. Propolis<br>2. HBSS<br>3. Aloe vera<br>4. Pomegranate juice
Nature´s benefaction as a life saver for an avulsed tooth	In Vitro Study	Saumya Navit, Niharika Shahi, Suleman Abbas Khan, Anshul Sharma, Vartika Singh, Ratna Priya Mishra, Pragati Navit, Prerna Sharma	2017	48 teeth	1. HBSS<br>2. Coconut water<br>3. Aloe vera<br>4. Saline	N/A	In order from most viable cells to the least<br>1. HBSS<br>2. Coconut water<br>3. Aloe vera<br>4. Saline<br><br>HBSS is the most effective storage media in maintaining PDL cell viability.

The comparative analysis of the effectiveness of four different storage media (Placentrex, propolis 10%, pomegranate juice 5%, and Hanks balanced salt solution) in preserving the viability of periodontal ligament cells	In Vitro Study	Musaffar Thoyalil, Dhanya Kamalakshan Belchada, Konsam Bidya Devi, Rekha Vasantha Ravi, Mridhul Madathikandy Uchummal, Ramnesh Parikkal	2023	60 teeth	1. HBSS<br>2. Placentrex<br>3. Propolis 10%<br>4. Pomegranate juice 5%	N/A	In order from most viable cells to the least<br>1. HBSS<br>2. Placentrex<br>3. Pomegranate juice<br>4. Propolis<br><br>All the other storage medias study were significantly inferior to HBSS

Metanalysis: Model 1 (HBSS vs Other Solutions)

Table S4: Final input of data for model 1 meta-analysis.

	TX			Control		
**AUTHOR**	nTX	mTX	sTX	nCT	mCT	sCT
Sagare et al. 2023	15	84.30	12.50	30	76.61	13.92
Abushana et al. 2022	20	38.48	2.32	60	25.35	2.43
Saini et al. 2017	23	144.79	14.40	46	76.00	12.39
Shetty et al. 2019	10	76800.00	4727.15	10	79072.00	7570.25
Abraham et al. 2019	10	921.40	608.44	20	526.85	321.87
Samreen et al. 2024	8	79.88	2.69	16	79.94	5.64
Babaji et al. 2017	10	262.00	3.13	30	241.67	3.65
Navit et al. 2017	12	87.33	5.24	36	67.01	4.39
Thoyalil et al. 2023	15	73.12	7.41	45	60.96	11.47

n=number of teeth; m=mean; s=SD; TX=Test HBSS group; CT=Other groups

Figure S1: Forest Plot for Model 1

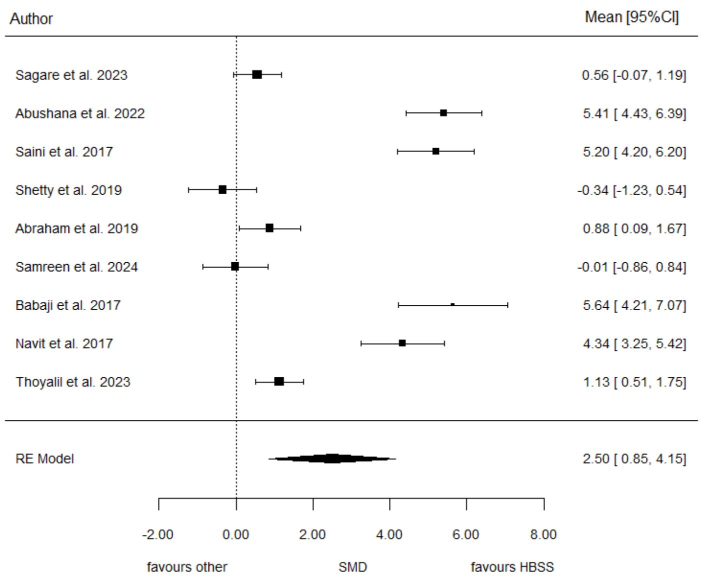

Figure S2: Funnel Plot for bias of publications in Model 1

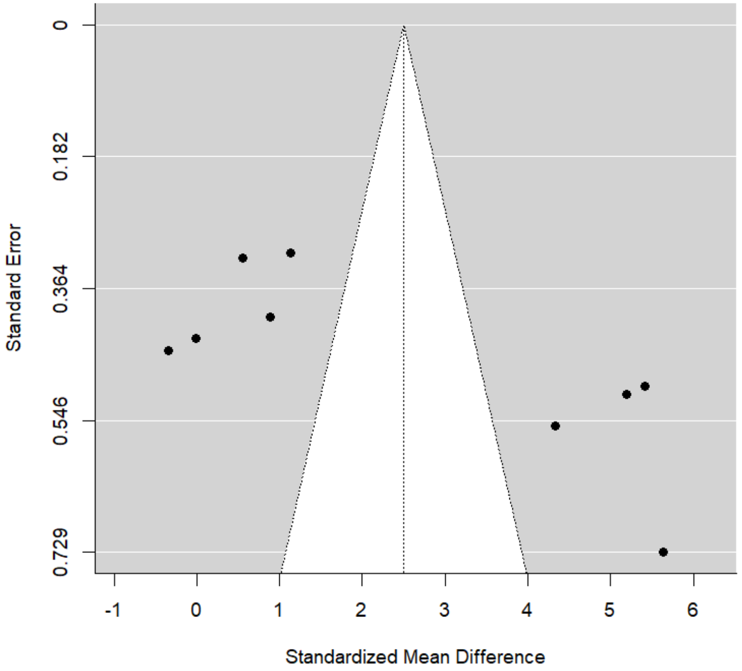

Table S5: Model 1 results of meta-analysis of standardized mean differences of PDL counts: mean difference (SMD) HBSS-Other, standard error (SE), 95% confidence interval, z test (p-value), I^2 index, Cochran´s Q statistic (p-value) for heterogeneity; Egger´s test (p-value) for publication bias

SMD	SE	95% CI		z (p-value)	I^2	Q_H (p-value)	Egger (p-value)
2.50	0.84	0.85	4.15	**0.003****	97.0%	<0.001***	0.004**

*p<0.05; **p<0.01; ***p<0.001

Figure S3: Galbraith´s Plot for Model 1

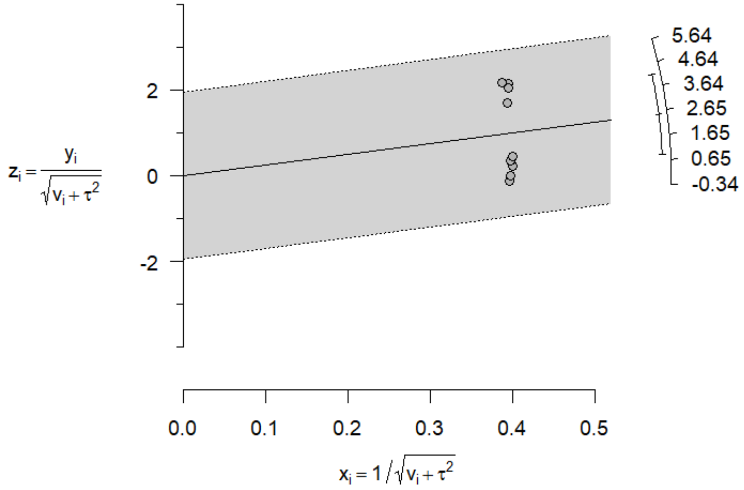

Figure S4: Funnel Plot for bias of publications in Model 1

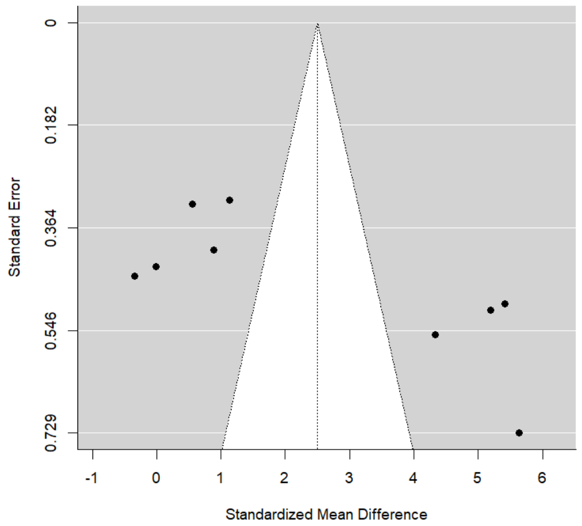

Meta-analysis: Model 2 (HBSS vs. Aloe Vera)

Table S6: Final input of data for model 2 meta-analysis.

	TX			Control		
**AUTHOR**	nTX	mTX	sTX	nCT	mCT	sCT
Abushana et al. 2022	20	38.48	2.32	20	30.36	1.86
Abraham et al. 2019	10	921.40	608.44	10	241.00	194.57
Babaji et al. 2017	10	262.00	3.13	10	226.00	3.43
Navit et al. 2017	12	87.33	5.24	12	70.59	4.73

n=number of teeth; m=mean; s=SD; TX=Test HBSS group; CT=Other groups

Figure S5: Forest Plot for Model 2

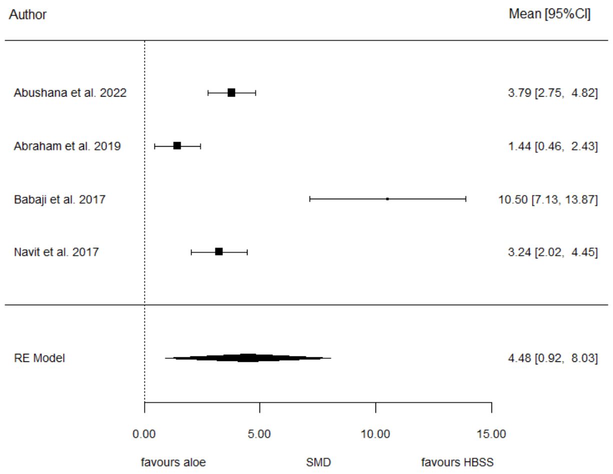

Table S7: Model 2 results of meta-analysis of standardized mean differences of PDL counts: mean difference (SMD) HBSS-Aloe, standard error (SE), 95% confidence interval, z test (p-value), I^2 index, Cochran´s Q statistic (p-value) for heterogeneity; Egger´s test (p-value) for publication bias

**SMD**	**SE**	**95% CI**	**z (p-value)**	**I^2**	**Q_H (p-value)**	**Egger (p-value)**
4.48	1.81	0.92 8.03	**0.014***	96.7%	<0.001***	<0.001***

*p<0.05; **p<0.01; ***p<0.001

Figure S6: Galbraith´s Plot for Model 2

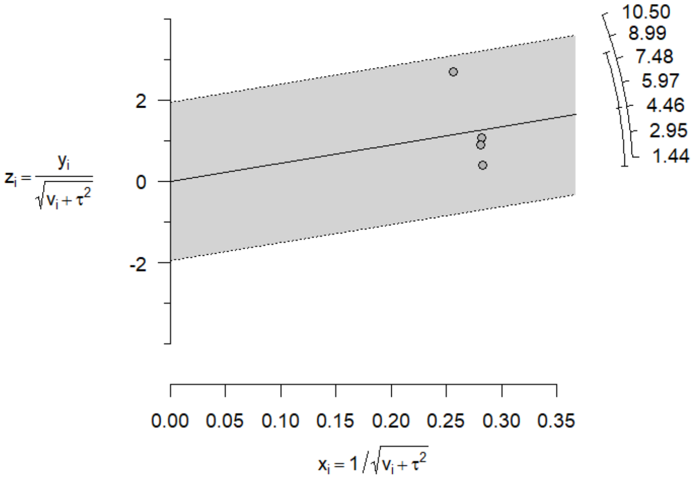

Figure S7: Funnel Plot for bias of publications in Model 2

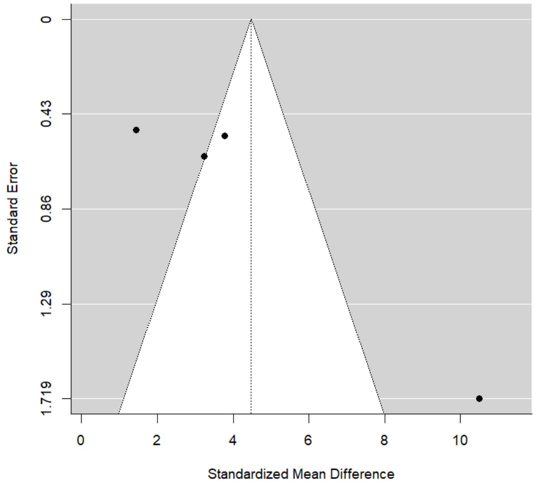

Table S8: Search results and exclusion reasons in the process of assessing the reports for eligibility

**EBSCO**				
11 results	Article	Author	Year	Reason for inclusion/exclusion
1	In vitro evaluation of storage media compared to HBSS for avulsed teeth:	Chakravorty, Ayushma	2024	EXCLUDE<br>Systematic review (part of exclusion criteria)
2	Evaluation of periodontal ligament cell viability in different storage media based on human PDL cell culture experiments	Osmanovic, Ahmed; Halilovic, Sabina; Kurtovic-Kozaric, Amina; Hadziabdic, Naida; Kurtovic-Kozaric, Amina.	2018	EXCLUDE<br>Systematic review (part of exclusion criteria)
3	Coconut milk and probiotic milk as storage media to maintain periodontal ligament cell viability: an in vitro study.	Saini, Divya; Gadicherla, Prahlad; Chandra, Prakash; Anandakrishna, Latha.	2017	INCLUDE
4	Potential of coconut water and soy milk for use as storage media to preserve the viability of periodontal ligament cells: an in vitro study	Moura, Camilla Cristhian Gomes; Soares, Priscilla Barbosa Ferreira; Paula Reis, Manuella Verdinelli; Fernandes Neto, Alfredo Júlio; Zanetta Barbosa, Darceny; Soares, Carlos José.	2013	EXCLUDE<br>Conducted on dog premolar teeth, and year of publication is greater than 10 years (part of exclusion criteria)
5	Evaluation of conditioned medium from placenta-derived mesenchymal stem cells as a storage medium for avulsed teeth: An in vitro study	Ji, Ling-Li; Song, Ge; Jiang, Li-Ming; Liu, Yao; Ding, Zhen-Jiang; Zhuang, Xue-Ying; Chen, Xu	2020	EXCLUDE<br>Measures only cell apoptosis not PDL viability following storage
6	Comparative evaluation of the clonogenic capacity of periodontal ligament fibroblasts in Hank’s balanced salt solution and egg albumen: An in vitro study	Shah, Dimpi; Sulkshane, Prasad; Lalwani, Rutika; Pawar, Sagar; Teni, Tanuja; Kakade, Adesh.	2018	EXCLUDE<br>Compares the clonogenic capacity not PDL cell viability following storage
7	Effect of storage temperature on the viability of human periodontal ligament fibroblasts.	Chen, Fubo; Qi, Shengcai; Lu, Liyan; Xu, Yuanzhi.	2015	EXCLUDE<br>Compares the effect of temperature of the storage mediums on PDL viability.
8	An in vitro evaluation of ice apple water, Aloe vera , and propolis as a storage medium to preserve viability of human periodontal ligament fibroblasts.	Bijlani S; Shanbhog R; Godhi BS; Talwade P; Tippeswamy HM	2022	EXCLUDED<br>Did not use HBSS as a media for comparison (HBSS is needed as a storage medium to be able to compare its effectiveness to other solutions in the same in vitro study)
9	Assessment of the viability of human periodontal ligament cells in black tea, lime juice, and passion fruit concentrate – A comparative in vitro study.	K. Deepthi1, Bikash Jyoti Borthakur2, B. Swathika3, S. Ganesan	2020	EXCLUDED<br>Did not use HBSS as a media for comparison (HBSS is needed as a storage medium to be able to compare its effectiveness to other solutions in the same in vitro study)

10	Evaluation of the efficacy of neem (Azadirachta indica) and turmeric (Curcuma longa) as storage media in maintaining periodontal ligament cell viability: An in vitro study	Dhimole, Pranjali; Bhayya, Deepak P.; Gupta, Shilpi; Kumar, Prabhat; Tiwari, Saurabh; Pandey, Swarnam.	2020	EXCLUDED<br>Did not use HBSS as a media for comparison (HBSS is needed as a storage medium to be able to compare its effectiveness to other solutions in the same in vitro study)
11	An in vitro evaluation of efficacy of ViaSpan, aloe vera, Gatorade solution, and propolis storage media for maintaining the periodontal ligament cell viability.	Misurya R; Sharma S; Syed Ismail PM; Gupta N; Rajan R; Kaur R; Babaji P	2022	EXCLUDED<br>Did not use HBSS as a media for comparison (HBSS is needed as a storage medium to be able to compare its effectiveness to other solutions in the same in vitro study)

**Web of Science**				
7 results	Article	Author	Year	Reason for inclusion/exclusion
1	Which is the most recommended medium for the storage and transport of avulsed teeth?	Adnan, Samira; Lone, Maham M.; Khan, Farhan R.; Hussain, Syeda M.; Nagi, Sana E.	2018	EXCLUDED<br>Systematic review (part of exclusion criteria)
2	The Comparative Analysis of the Effectiveness of Four Different Storage Media (Placentrex, Propolis 10%, Pomegranate Juice 5%, and Hank's Balanced Salt Solution) in Preserving the Viability of Periodontal Ligament Cells: An In Vitro Study	Thoyalil, Musaffar; Belchada, Dhanya Kamalakshan; Devi, Konsam Bidya; Ravi, Rekha Vasantha; Uchummal, Mridhul Madathikandy; Parikkal, Ramnesh	2023	INCLUDED
3	Effects of tooth storage media on periodontal ligament preservation	Hasan, Md Riasat; Takebe, Hiroaki; Shalehin, Nazmus; Obara, Nobuko; Saito, Takashi; Irie, Kazuharu	2017	EXCLUDED<br>Study was carried out on rats (part of exclusion criteria)
4	Efficacy of Natural Coconut Water, Pre-packaged Coconut Water, and Hank's Balanced Salt Solution as Storage Media in Maintaining Periodontal Ligament Cell Viability: An In-vitro Study	Sara, Samreen; Kesri, Rituraj; Ukey, Ankita; Surana, Pratik; Sahu, Anshuta; Agrawal, Pankaj; Owais, Rahman	2024	INCLUDED
5	An In Vitro Evaluation of Morinda citrifolia and Ocimum sanctum as Potential Storage Media to Maintain Cell Viability for Avulsed Teeth Using Collagenase Dispase Assay	Sagare, Shweta Vijaykumar; Patil, Anand; Patil, Pranav; Kumar, R. Susheel; Gangishetti, Sairam; Ingale, Priya	2023	INCLUDED

6	Comparative Evaluation of The Efficacy of Aloe Vera Gel with Milk and Hank's Balanced Salt Solution in Maintaining the Viability of PDL Cells in Avulsed Teeth	Abraham, Baren; Kumaran, Parvathy; Varma, B. R.; Xavier, Arun Mamachan; Kumar, Suresh J.	2019	INCLUDED
7	Survival of human periodontal ligament fibroblast cells in Cornisol and HBSS for transportation of avulsed teeth: a comparative ex vivo study	Singh, Shruti; Kini, Sandya; Pai, Swathi; Rajeshwari, R. H.; Purayil, Tina Puthen	2021	EXCLUDED<br>Study was conducted ex-vivo (part of exclusion criteria)

**SCOPUS**				
9 results	Article	Author	Year	Reason for inclusion/exclusion
1	Assessment of the Efficacy of Different Storage Media for Maintaining an Avulsed Tooth: An In Vitro Study	AlWaleed Abushanan, Abdulfatah Alazmah, Uthman S Uthman, Adel S Alqarni, Abdulhamid Al Ghwainem, Narendra Varma Penumatsa	2022	INCLUDED
2	Comparative Evaluation of Efficacy of Platelet-Rich Fibrin and Hank’s Balanced Salt Solution as a Storage Medium for Avulsed Teeth: An In Vitro Study	Ashwija Shetty, Somnath Gnosh, A Srirekha, T Jaykumar, Champa Chikkamallaiah, Savitha Adiga	2019	INCLUDED
3	Comparative in vitro study of the effectiveness of Green tea extract and common storage media on periodontal ligament fibroblast viability	Fahimeh Adeli , Ebrahim Zabihi , Zeinab Abedian , Samane Gharekhani , Mahdi Pouramir , Soraya Khafri , Maryam Ghasempour	2019	EXCLUDED<br>No data was provided on the number of PDL cells that were viable after storage
4	In vitro comparative evaluation of different storage media (hank’s balanced salt solution, propolis, Aloe vera, and pomegranate juice) for preservation of avulsed tooth	Prashant Babaji, Mahesh Melkundi, Raghu Devanna, Suresh B.S., Vishwajit Rampratap Chaurasia, Gopinath P.V	2019	INCLUDED
5	Potential of coconut water and soy milk for use as storage media to preserve the viability of periodontal ligament cells- an in vitro study	Camilla Cristhian Gomes Moura, Priscilla Barbosa Ferreira Soares, Manuella Verdinelli de Paula Reis1, Alfredo Julio Fernandes Neto, Darceny Zanetta Barbosa, Carlos Jose Soares	2014	EXCLUDED<br>The study was conducted on dog premolar teeth (part of exclusion criteria)
6	In Vitro Periodontal Ligament Cell Viability in Different Storage Media	Meenakshi Sharma	2016	EXCLUDED<br>Did not use HBSS as a media for comparison (HBSS is needed as a storage medium to be able to compare its effectiveness to other solutions in the same in vitro study)

7	Comparative in vitro study of the effectiveness of Green tea extract and common storage media on periodontal ligament fibroblast viability	Fahimeh Adeli , Ebrahim Zabihi , Zeinab Abedian , Samane Gharekhani , Mahdi Pouramir , Soraya Khafri , Maryam Ghasempour	2019	EXCLUDED<br>Did not use HBSS as a media for comparison (HBSS is needed as a storage medium to be able to compare its effectiveness to other solutions in the same in vitro study)
8	Effect of milk renewal on cell viability in vitro at different time frames	Beatriz Dulcineia Mendes de Souza, Ana Maria Hecke Alves, Dayane Machado Ribeiro, Luciane Geanini Pena dos Santos, Claudia Maria de Oliveira Simões, Wilson Tadeu Felippe, Mara Cristina Santos Felippe	2017	EXCLUDED<br>Did not use HBSS as a media for comparison (HBSS is needed as a storage medium to be able to compare its effectiveness to other solutions in the same in vitro study)
9	Natures benefaction as a life saver for an avulsed tooth: an in vitro study	Saumya Navit, Niharika Shahi, Suleman Abbas Khan, Anshul Sharma, Vartika Singh, Ratna Priya Mishra, Pragati Navit, Prerna Sharma	2017	INCLUDED
